# Pleistocene Forest Stability Predicts Patterns of Frog Diversity in Central Africa

**DOI:** 10.1002/ece3.73207

**Published:** 2026-03-11

**Authors:** Gregory F. M. Jongsma, Narayani Barve, Julie M. Allen, Hannah L. Owens, David C. Blackburn

**Affiliations:** ^1^ Department of Natural History New Brunswick Museum Saint John Canada; ^2^ Florida Museum of Natural History University of Florida Gainesville Florida USA; ^3^ Department of Biological Sciences Virginia Tech Blacksburg Virginia USA; ^4^ Center for Macroecology, Evolution, and Climate, Globe Institute University of Copenhagen Copenhagen Denmark; ^5^ Center for Global Mountain Biodiversity, Globe Institute University of Copenhagen Copenhagen Denmark

**Keywords:** Afrobatrachia, amphibian, conservation, historical biogeography, phylogenetic, refugia

## Abstract

Understanding why diversity is unevenly distributed across the globe is central to ecology and evolution. Focusing on Afrobatrachian frogs of the Lower Guinean Forests of western Central Africa, we combined Pleistocene‐to‐present palaeoclimatic reconstructions with ecological niche models to delineate areas of long‐term habitat stability (“refugia”). We tested whether refugia or contemporary climate best explain present‐day patterns of species richness, phylogenetic diversity, and weighted endemism. Strict and relaxed stability thresholds identified core refugia that occupy only a small fraction of the landscape yet harbor the highest values for each diversity metric. Spatial regression models revealed that past forest stability accounted for 65%–71% of the spatial variance in richness and phylogenetic diversity, far exceeding the explanatory power of current abiotic conditions, and that all diversity metrics declined sharply with increasing distance from the edge of stable forest. Because less than 15% of inferred refugia fall within existing protected areas, expanding conservation coverage to include the most stable forest blocks would increase mean amphibian richness in protected areas by nearly one‐fifth. Our results demonstrate that the legacy of Pleistocene forest dynamics outweighs contemporary climate in shaping amphibian diversity within this tropical hotspot. We provide a transferable, quantitative workflow for integrating past habitat stability into regional conservation planning.

## Graphical abstract

Using high‐resolution paleoclimate reconstructions, we quantified habitat stability for Central African rainforests across 257 time slices since the early Pleistocene. Stacked projections of forest‐dependent frog niches identify both a large, continuous refugium and smaller, highly stable forest blocks embedded within today's forest cover. These stability maps provide an explicit baseline for evaluating how past forest dynamics shape present‐day patterns of amphibian richness and endemism, which can.

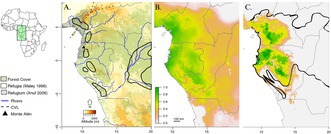

## 1 Introduction

**FIGURE 1:**
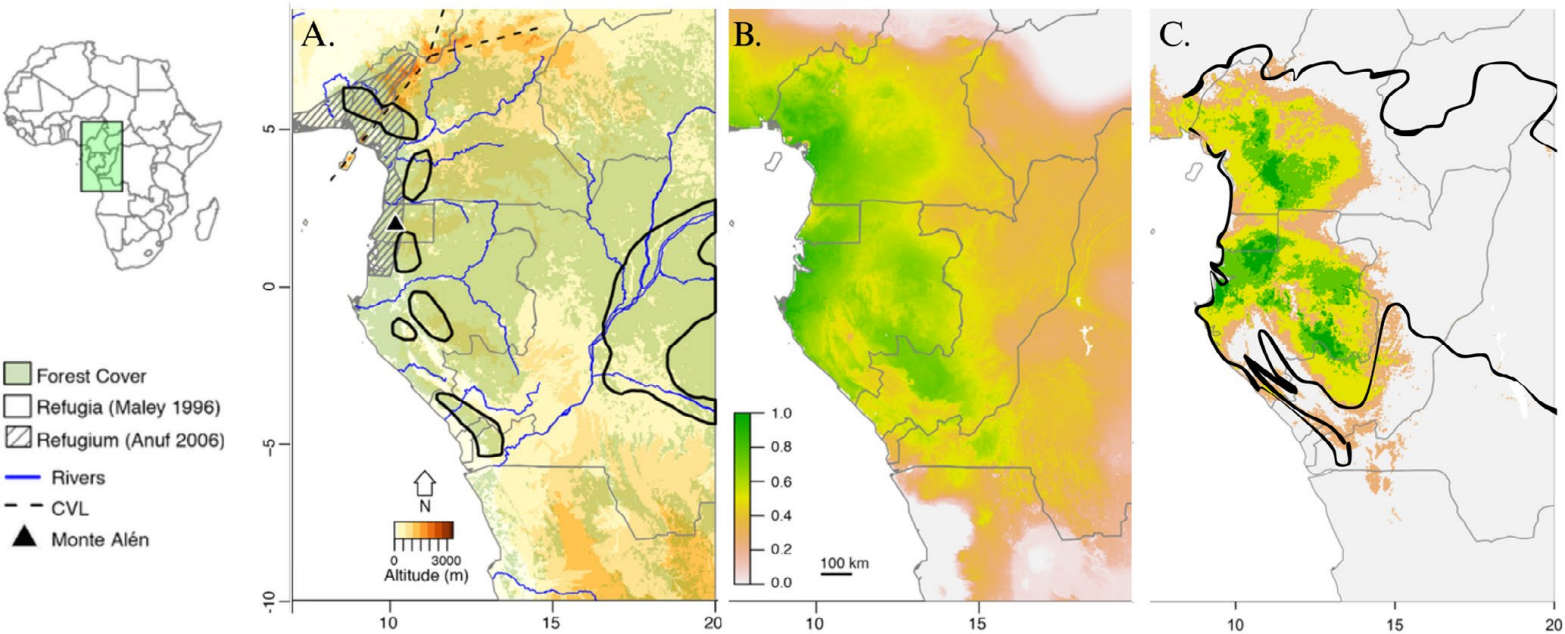
(A) Previously delimited refugia (Maley 1996; Anhuf et al. 2006), contemporary forest, rivers, and two geographic centers of endemism (Cameroon Volcanic Line [CVL] and Monte Alén). Refugia polygons were georeferenced in QGIS (v3.16) using their original publications. (B) Pleistocene forest stability map based on 10 forest‐obligate non‐afrobatrachian frog species, created by stacking the thresholded ENM models for all species for all 257 time points. The darker green shows areas with higher suitability through time, and white indicating areas that were never suitable. (C) Pleistocene forest stability map for different thresholds (95% dark green; 90% light green; 75% yellow; 50% beige), which were used to measure changes in diversity with distance from their edges. Black outline represents the extent of contemporary forest cover.

The uneven distribution of species across the globe is one of the oldest observed patterns in biology (Lomolino 2004). Many hypotheses attempt to explain higher diversity in the tropics through ecological or evolutionary mechanisms (reviewed in Fine 2015). Most ecological hypotheses rely on contemporary climate or net primary productivity to explain heightened species diversity in the tropics (Wright 1983; O'Brien 1998). Advocates of contemporary climate hypotheses expect that species richness is in close equilibrium with the current climate as species' distributions quickly respond to changing environments (Hawkins et al. 2003). Evolutionary hypotheses emphasize the role of past environmental stability in influencing contemporary patterns of species diversity by promoting long‐term persistence and the accumulation of lineages through time, resulting in a pattern of fewer taxa in areas with low stability (McGlone 1996; Jablonski et al. 2006; Wiens et al. 2009). Areas of past habitat stability (typically conceptualized as “refugia”) are among the oldest hypothesized explanatory variables of both species distributions (Forbes 1846) and areas of speciation (Haffer 1969). Because most studies have largely relied on present‐day patterns of species diversity to infer postulated areas of stability, these refugia are usually represented in binary terms (present or absent; Figure 1B). To disentangle the ecological and evolutionary processes that explain contemporary biodiversity, we need rigorous and quantitative estimates of climate and habitat stability, which are now possible using paleoclimatic models and ecological niche modeling (Araújo et al. 2008; Silva et al. 2024).

The recent development of global paleoclimate and current habitat models makes it feasible to test the roles of contemporary conditions and past stability in a statistical framework. Quantitative contemporary climate data are readily available from the WorldClim database (Hijmans et al. 2005). However, producing quantitative stability data is much less straightforward and has impeded comparisons between past and contemporary climates (Graham et al. 2006). Evidence of climate stability is typically measured by examining the anomaly between a past time period (usually the Last Glacial Maximum; LGM) and contemporary climate (Jansson 2003; Colville et al. 2020). In such analyses, areas with the least change over time are considered more stable. Because climate anomalies represent the difference between past and present climates, past and contemporary variables tend to have high correlation and collinearity. Therefore, it may be difficult to identify the true mechanism explaining diversity (Araújo et al. 2008). Such estimates of climate stability are potentially blind to whether an area is stable but unsuitable for a specific organism. For example, areas in a study region can be equally stable for extreme conditions, such as high or low precipitation, for which organisms with strict physiological requirements could have dramatically different responses. Although incorporating quantitative measures of climate stability for testing competing biogeographic hypotheses is increasingly popular (Araújo et al. 2008; Colville et al. 2020; Saladin et al. 2020), quantitative measures of habitat stability remain rare (Graham et al. 2006; Rosauer et al. 2015; Barratt et al. 2017). Ecological Niche Modeling (ENM) offers an alternative approach for estimating the stability of suitable habitats relevant to specific taxa.

The Lower Guinean Forest (LGF) in equatorial Africa is an ideal region for testing the influence of past versus contemporary drivers of tropical diversity due to its rich biodiversity, variable climate, and the existence of several postulated areas of habitat stability (e.g., forest refugia). Amphibian communities in the LGF are characterized by high levels of forest specialization and endemism, making them particularly sensitive to changes in forest structure and microclimate (Ernst et al. 2006). Across Central Africa, habitat loss and degradation driven by logging, mining, and agriculture represent the dominant threats to amphibians, with ongoing climate change expected to exacerbate these pressures furthur (Rödel et al. 2021; Clements et al. 2025). The LGF extends from eastern Nigeria, across Cameroon, south through Gabon and the Republic of Congo to the southern Democratic Republic of Congo, and extends east to the Ubangi River and the Congo River (Figure 1; White and Unesco 1983; Hardy et al. 2013). The LGF was initially identified as a single, large refugium based on patterns of endemism in animals and plants (Carcasson 1964; Endler 1982; White and Unesco 1983; Mayr and O'Hara 1986; Prigogine 1988) and later by paleoprecipitation models (Anhuf 2000; Anhuf et al. 2006). However, estimating forest refugia based only on patterns of contemporary endemism and richness runs the risk of being circular in reasoning (Hamilton et al. 2001). Maley (1987, 1996) identified five smaller refugia within the LGF using a hybrid approach combining three lines of evidence: (a) the pollen record, which supports climate‐driven vegetation shifts (Livingstone 1975; Maley and Livingstone 1983), (b) patterns of plant and animal endemism, and (c) elevation, considering that mountains cause upward airflow, resulting in increased moisture and rainfall (Maley 1996). However, most bodies of standing water in Central Africa with sediments suitable for sampling pollen are young (~15,000 years kry). This makes it challenging to map the vegetation of the region earlier than the Late Pleistocene in detail (Livingstone 2001; Anhuf et al. 2006). While there is a long history of interest in the role of habitat stability in the LGF (Carcasson 1964; reviewed in Couvreur et al. 2021), no studies have directly compared contemporary and past variables as predictors of modern diversity in this biodiversity hotspot. Paleoclimate models offer promising new avenues for exploring past forest dynamics deeper in time than the palynological records allow in Central Africa.

Afrobatrachia (Frost et al. 2006) is a species‐rich clade of frogs endemic to sub‐Saharan Africa within the suborder Neobatrachia, for which the LGF is a center of diversity. In this study, we compare the relative influence of contemporary and past variables in shaping present‐day patterns of diversity in this clade. As a proxy for past forest stability, we developed a quantitative estimate of long‐term habitat suitability based on ENMs of forest‐obligate, non‐afrobatrachian frog species. This provides an estimate of forest stability that is independent of the diversity patterns of our focal clade and allows direct comparison of past stability with contemporary environmental metrics. We used spatially explicit regression models to test the relative influence of each variable on four measures of diversity. To confirm the role of stable forest refugia, we evaluated whether diversity decays with increasing distance from the nearest refugium. If species are at or near equilibrium, as expected under ecological hypotheses that emphasize contemporary climate patterns, diversity is expected to be constant across current forest cover. We anticipate that developing quantitative measures of past habitat stability based on high‐resolution paleoclimate models will provide a powerful inference tool to test other hypotheses about the distribution of contemporary biodiversity more widely and help inform effective conservation actions.

## 2 Methods

### 2.1 Diversity Metrics

We explored contemporary patterns of species diversity in the LGF using Afrobatrachia as a model. Afrobatrachia includes four families, three of which occur in Central Africa (Arthroleptidae, Hemisotidae, and Hyperoliidae); the fourth, Brevicipitidae, is restricted to southern and eastern Africa. Within our study region (Figure 1), there are ~127 species in 17 genera, of which we included 124 species from 16 genera; three species were not included because they have no sequence data available and no hypothesized sister taxa. The study area was divided into 4.5 × 4.5 km cells, which we will refer to as sampling units (SUs). We produced four diversity metrics with these data: species richness (SR; Colwell 2009), phylogenetic diversity (PD; Faith 1992), relative phylogenetic diversity (RPD; Mishler et al. 2014), and phylogenetic endemism (PE; Rosauer et al. 2015). The metrics were chosen based on their potential to provide insight into processes driving diversification or for conservation planning strategies. Species richness (SR), the simplest metric, is the number of species found in each SU, which was derived by stacking all species distribution maps. Phylogenetic diversity (PD) is the summed evolutionary history (branch‐length) of all the species in an SU, based on the phylogenetic tree of the species present. Relative PD (RPD) is the ratio of the PD measured on the original phylogenetic tree divided by the PD measured on a theoretical tree whose branches are of equal length. These relative metrics are designed to identify areas of overdispersion and clustering, which may reflect the gradual accumulation of lineages or recent radiations, respectively (Mishler et al. 2014). Phylogenetic endemism (PE) of a given grid cell is the sum of branch lengths (the root of the phylogeny to the tip), weighted by the inverse of the branch's range (total grid cells occupied by the species), for each species present (Rosauer et al. 2015). All metrics are reported between 0 and 1, with 1 being the highest.

The calculation of the diversity metrics used here requires distribution maps for each species. Distributions of many African species remain coarsely delimited, and existing maps from IUCN (2018) are often only a general approximation of distribution, especially for widespread species. We generated ENMs for all species with sufficient unique records (> 10; a commonly used threshold to ensure model reliability; van Proosdij et al. 2016), using maximum entropy as implemented in Maxent (v3.3; Phillips et al. 2017; full details of variable selection and model calibration are provided in the Supporting Information S1). Models with average AUC ≤ 0.70 were excluded, and projections were restricted to a 100 km buffer around the IUCN range map. We used IUCN (2018) maps for species that are microendemics or had too few records to produce reliable models. Our resulting ENMs produced fine‐scale patterns not possible with IUCN maps alone, primarily because of increased sampling efforts in Gabon, Republic of Congo, and northern Angola over the past two decades (Burger et al. 2004; Dewynter et al. 2018; Ernst et al. 2020; Jackson and Blackburn 2010; Jongsma et al. 2017). We built on a previous well‐resolved phylogeny of Afrobatrachia (Portik et al. 2019), adding missing taxa using the mitochondrial ribosomal *16S* locus from GenBank and our unpublished sequences (15 taxa; Table S1). We constrained the topology of our phylogeny to that of Portik et al. (2019), which was based on > 1000 loci, using the “‐g” function in RaxML (v.8). Three species ( *Leptodactylodon blanci* , *L. stevarti* , and *Hyperolius bopeleti* ) that had no sequence data available were added to the phylogeny based on their hypothesized relationships using the *add.tip* function in “Phylotools” (v.0.7–70). Their branch lengths were estimated by calibrating the age of the sister species or species group to which they likely belong (Figure S1).

The phylogeny and spatial data were combined in the program Biodiverse v3.1 (Laffan et al. 2010) to calculate SR, PD, RPD, and PE metrics for each SU (4.5 × 4.5 km resolution grids). The SR, PD, and RPD maps were constrained to a bounding box containing the LGF and surrounding area (upper left: 12° N, 7° E; lower right: 10° S, 20° E). To calculate the range size metrics for species with distributions that extend beyond our region of interest, the map used to calculate PE spanned all of Africa.

### 2.2 Diversity Predictors

Correlates of diversity were measured for three uncorrelated contemporary variables (temperature, precipitation, forest tree density) to test the hypothesis that species are in equilibrium with present‐day environmental conditions. We downloaded annual mean temperature (BIO1) and annual mean precipitation (BIO12) from WorldClim.org at 2.5 arc minute resolution. Forest density was quantified as tree stem density (number of trees per km^2^) using the global tree density product of Crowther et al. (2015), which estimates the spatial distribution of trees ≥ 10 cm diameter at breast height based on extensive field plot data and biome‐specific models. Climate stability was estimated for mean annual temperature (BIO1) and precipitation (BIO12) across 257 interpolated time‐slices spanning from 2.58 million years ago (myr) to 20,000 years ago (kyr; Gamisch 2019). First, we calculated the mean standard deviation of each climate variable (BIO1, BIO12) between time points over the time elapsed converted to stability, defined as the inverse of this deviation (Owens and Guralnick 2019). To discriminate between the effects of contemporary and past variables (Araújo et al. 2008), we did not include present‐day values in our calculations of temperature and precipitation stability but instead used time‐slices from 2.58 myr to 20 kyr. Raw spatial layers for predictor variables used in analyses are shown in Figure S3.

We estimated forest stability since the Pleistocene using ENMs for 10 frog species that are not members of the Afrobatrachia and thus independent of our diversity metrics (Table S2). We selected these 10 species in six genera (*Amnirana*, *Chiromantis*, *Conraua*, *Phrynobatrachus*, *Ptychadena*, and *Sclerophrys*) because they are common, forest‐obligate species that occupy different microhabitats (arboreal, terrestrial, aquatic), and are largely co‐distributed across Central Africa (details in Table S2). We calculated the contemporary niche for each of these 10 species using presence records and Maxent (v3.3; Phillips et al. 2017; details in Supporting Information S1), and projected these models back in time to reveal similar environmental conditions at each past time point (2.58 myr–20 kyr at 10 kyr time intervals). A 95% minimum occurrence point threshold was applied to each species' model at each time interval, which uses the 5th percentile suitability score to classify the continuous Maxent suitability scores across the study area into suitable versus unsuitable habitat. Finally, we summed models over all time periods for all species to create a final spatio‐temporal estimate of past forest stability. Additionally, we applied thresholds to our forest stability map to create qualitative delimitations of stability, for which we use the term “refugia,” that were required for evaluating the relationship of diversity with distance from refugia. The strictest refugia were defined as having 95% of our forest stability indicator species present across 230 or more time‐slices, and again for 90%, 75%, and 50% of species present. Each cell that met a specific threshold was converted to “one” and cells that did not were converted to “zero.”

### 2.3 Spatial Analyses

To measure the relative influence of contemporary and past variables on diversity, we ran a series of univariate and multivariate regression models. Prior to model fitting, we assessed collinearity among predictors using pairwise Pearson correlation coefficients; correlations were weak to moderate (|*r*| ≤ 0.69), indicating no problematic multicollinearity. Using the *lm* function in R (v4.0.2), we ran individual ordinary least squares (OLS) regressions for each of the four diversity metrics (SR, PD, RPD, PE) as the dependent variables and each of the contemporary and past environmental variables as the predictors. Next, we created two multivariate regressions for each diversity metric, combining the three contemporary variables as one model and the past variables as a second model. To account for spatial autocorrelation, which can inflate Type I errors (Dormann et al. 2007), we also ran these analyses using the “spatial lag of X” model (SLX). To test the best mixed model, including all possible variables, for explaining the different diversity metrics, we ran stepwise regression analyses. Using the R package “olsrr” (Hebbali 2020), predictive variables were iteratively added to find the best subset of variables resulting in the best performing model based on AIC scores. Finally, to further tease apart the influence of past forest stability versus current forest cover (measured as tree density), we created a distance matrix using the *distance* function in the R package “raster” v3.4 (Hijmans and van Etten 2012) from the edge of our strictest refugia (calculated as areas that were suitable for 95% of species across 90% of the time‐slices; Figure S4). We ran OLS and SLX models with each diversity metric as the dependent variable and distance from the edge of the nearest refugium as the predictor variable.

### 2.4 Conservation

To calculate the proportion of past stable forest that is currently protected, we used the *gIntersection* function in the R package “rgeos” to measure the amount of overlap between our thresholded, binary refugia (Figure S4) with the World Database on Protected Areas (WDPA; UNEP‐WCMC and IUCN, 2021). Using the *extract* function in the R package “raster” v3.4 (Hijmans and van Etten 2012), we calculated the average mean richness per grid cell for each thresholded refugia category (defined as suitable for 50%, 75%, 90%, and 95% of species through time) for protected areas within these refugia categories as well as for all protected areas together. We also evaluated whether conserving the entirety of our strictest category of refugia (95% species threshold) would significantly increase the average number of species in protected areas overall. To do so, we calculated the mean richness for 1000 randomly sampled cells in three categories (protected areas [PA]; PA + 95% refugia; PA + random area [equal in area to 95% refugia]) and repeated this 100 times to generate an estimate.

## 3 Results

### 3.1 Diversity Metrics

**FIGURE 2:**
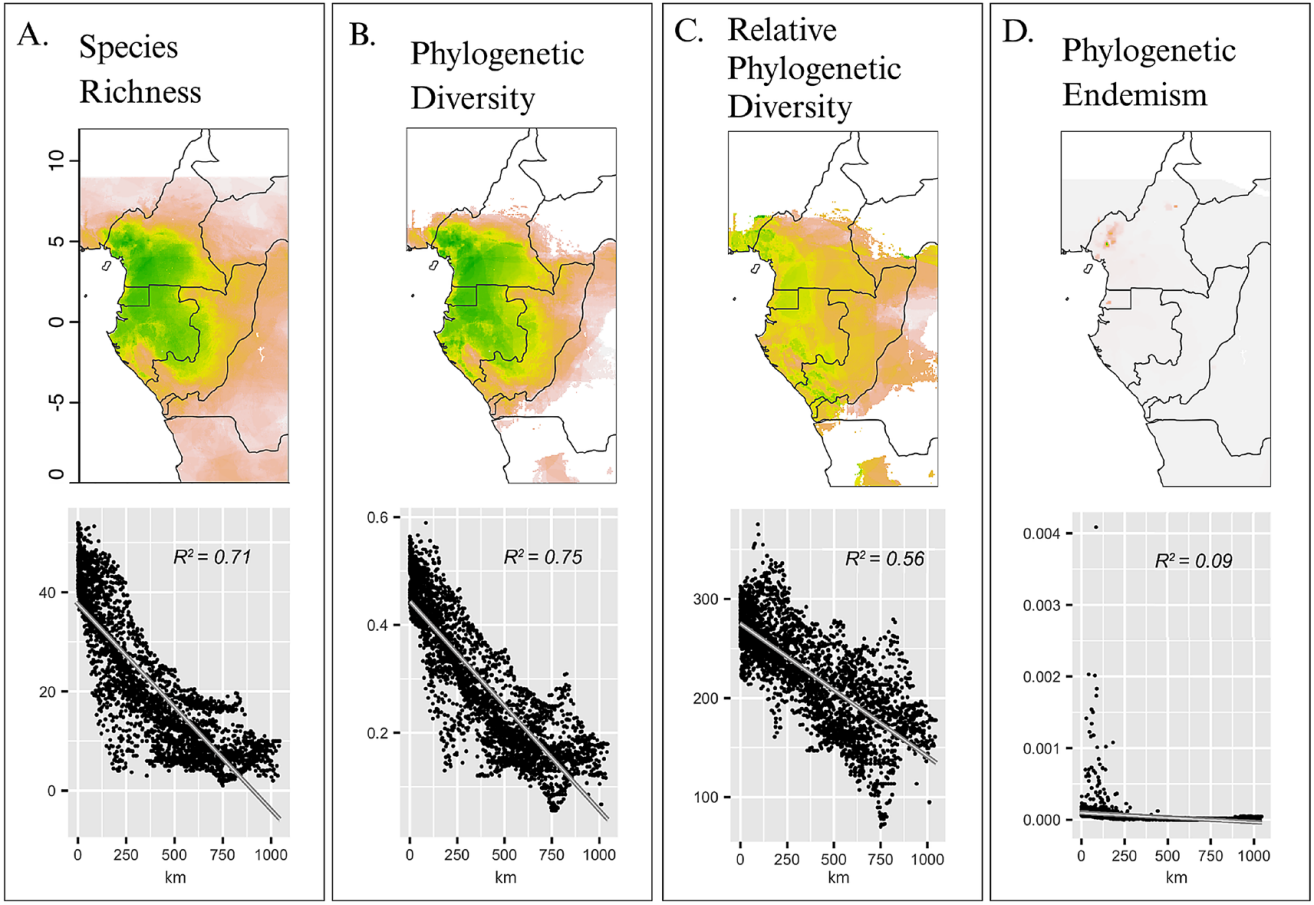
Diversity of afrobatrachian frogs decays with increasing distance from the edge of the nearest refugium. (A) Species richness, (B) Phylogenetic diversity (PD), (C) relative phylogenetic diversity (RPD), and (D) phylogenetic endemism (PE). Based on the edge of the 95% stability threshold (Figure 1C).

Our diversity metrics were based on 124 species of afrobatrachian frogs (Figure 2). We modeled the distributions of 84 of these species and used IUCN maps for the remaining micro‐endemics or undersampled species. This final dataset revealed high‐resolution (4.5 km grid cells) patterns of afrobatrachian diversity for the region. Species richness (SR), PD, and RPD are highest in forested regions of southern Cameroon, Equatorial Guinea, Gabon, and eastern Republic of the Congo (Figure 1). Phylogenetic endemism is concentrated in the Cameroon Volcanic Line (CVL) in western Cameroon and Monte Alén in Equatorial Guinea (Figure 2).

### 3.2 Forest Stability Estimates

The 50%–75% forest stability indicator species threshold supports the existence of one continuous, large refugium. It almost entirely overlaps with previous delimitations (Prigogine 1988; Mayr and O'Hara 1986; Anhuf 2000) but extends the area of stability further south through Gabon and the Republic of Congo with more fragmented patches of stability (50% threshold) extending as far as northern Angola (Figure 1). At stricter thresholds (90%–95% of species), we found a more fragmented landscape of stability, more in line with the refugia outlined by Maley (1987, 1996; Figure 1).

### 3.3 Spatial Analyses

Univariate ordinary least squares (OLS) and spatial lag of X (SLX) models show that our measure of forest stability is the best predictor of most metrics of afrobatrachian diversity (SR, PD, RPD; Figure S2). The one exception is PE, which was predicted equally well or slightly better by contemporary precipitation (Table 1). After forest stability, contemporary precipitation and contemporary forest (tree density) were consistently the next strongest predictors of the diversity metrics (Table 1). Past climatic stability (temperature and precipitation) and contemporary temperature were poor predictors (*R* ^2^ < 0.5) of diversity or else non‐significant (*p*‐value > 0.05).

**TABLE 1:** Standardized coefficients of predictors from univariate regressions (OLS: ordinary least squares and SLX: spatial lag of X spatial model) for the four diversity metrics.

		St. coefficient	OLS *R* ^2^	St. coefficient	SLX adjusted *R* ^2^
Richness	Temperature	−0.18	0.03	−0.17	0.03
	Precipitation	0.54	0.32	0.82	0.40
	Current forest	0.45	0.19	0.99	0.34
	Temp. stability	−0.11	0.01	−0.23	0.02
	Precip. stability	—	—	—	—
	Forest stability	0.80	0.65	0.97	0.71
Phylogenetic diversity	Temperature	−0.21	0.04	−0.20	0.04
	Precipitation	0.54	0.32	0.81	0.40
	Current forest	0.44	0.17	0.96	0.32
	Temp. stability	−0.15	0.02	−0.27	0.03
	Precip. stability	—	—	—	—
	Forest stability	0.82	0.69	1.01	0.76
Relative phylogenetic diversity	Temperature	−0.28	0.08	−0.26	0.075
	Precipitation	0.44	0.22	0.58	0.24
	Current forest	0.27	0.07	0.65	0.14
	Temp. stability	−0.22	0.05	−0.29	0.05
	Precip. stability	0.05	0.00	—	—
	Forest stability	0.73	0.54	0.88	0.60
Phylogenetic endemism	Temperature	−0.18	0.04	0.06	0.09
	Precipitation	0.25	0.09	0.34	0.10
	Current forest	0.12	0.02	0.21	0.02
	Temp. stability	−0.05	0.00	−0.14	0.01
	Precip. stability	—	—	0.11	0.01
	Forest stability	0.27	0.09	0.28	0.09

*Note:* Total explained variance (*R* ^2^) is also presented. Dependent variables: richness, species richness. Only effects that were significant (*p* < 0.05) are shown.

Abbreviations: PD, phylogenetic diversity; PE, phylogenetic endemism; RPD, relative phylogenetic diversity.

The results from our multivariate regressions revealed that the combined past variables better predict SR, PD, and RPD than the contemporary variables (Table 2). Variation in PE is better explained by contemporary variables (*R* ^2^ = 0.22 vs. *R* ^2^ = 0.10); however, much of this variation remains unexplained, suggesting there are other unaccounted drivers of PE in this region. The stepwise regression analysis, which tests for the best subset of predictor variables, consistently supported four variables: forest stability, precipitation, temperature, and contemporary forest (*R* ^2^ = 0.14–0.73). We reran these analyses, excluding forest stability, and found that the *R* ^2^ of the next best models never surpassed 0.43.

**TABLE 2:** Standardized coefficients and *R* ^2^ of predictors from multivariate regressions (OLS: ordinary least squares and SLX: spatial lag of X spatial model) for the four diversity metrics.

Diversity metric	Model	Contemporary				Past			
		Temp	Precip	Forest	*R* ^2^	Temp stability	Precip stability	Forest stability	*R* ^2^
Richness	OLS	−0.13	0.47	0.30	0.43	0.16	−0.06	0.84	0.67
PD	OLS	−0.16	0.48	0.27	0.42	0.11	−0.04	0.86	0.70
RPD	OLS	−0.26	0.42	0.10	0.30	−0.04	0.052	0.72	0.55
PE	OLS	−0.18	0.25	0.02	0.13	0.02	0.01	0.27	0.10
Richness	SLX	−0.13	0.48	0.35	0.45	0.16	—	0.86	0.68
PD	SLX	−0.17	0.50	0.31	0.45	—	—	0.88	0.72
RPD	SLX	−0.28	0.44	0.12	0.32	—	—	0.74	0.57
PE	SLX	−0.14	0.30	—	0.22	—	—	0.27	0.10

*Note:* Dependent variables: richness, species richness.

Abbreviations: PD, phylogenetic diversity; PE, phylogenetic endemism; RPD, relative phylogenetic diversity.

Because the areas of high forest stability are located within contemporary forests, we used distance from the nearest refugium to disentangle the role of past versus current forests on patterns of diversity. Even when restricting the analyses to contemporary forest cover, all of the diversity metrics declined with increasing distance from the edge of the nearest refugium (Figures 1 and 2).

### 3.4 Conservation

A relatively small percentage (12.5%–14.5%) of the strict (95% threshold) stable forest areas are currently protected. These stable areas are of high conservation importance because they contain, on average, higher species richness and phylogenetic diversity than protected areas taken as a whole for the region (Figure S5). Expanding protected areas to cover a broader extent of past stable forest would increase the average diversity of afrobatrachian frogs under protection in the region (mean richness increase from 23.1 ± 0.8 SD to 27.1 ± 0.9 per cell, an 18% increase; Figure S6).

## 4 Discussion

Most studies evaluating the relative roles of ecological and evolutionary processes on patterns of species richness focus on the latitudinal diversity gradient (Fine 2015; Jansson 2003; Jablonski et al. 2006). However, testing these hypotheses at such large scales can conflate two central processes—biome stability and productivity—that are both highest at the equator (Colville et al. 2020). By conducting our study at a regional scale, we can more effectively disentangle the primary processes driving contemporary patterns of biodiversity in this tropical system.

Our study demonstrates that forest stability since the Pleistocene is a strong positive predictor of afrobatrachian frog diversity in Central Africa. This further supports the importance of past stability in generating and maintaining diversity in the tropics. Although forest stability explained the largest proportion of variance in species richness and phylogenetic diversity, univariate regression models demonstrate that contemporary forest and precipitation contribute substantially to present‐day patterns of diversity (Table 1; Figure S2). We disentangled the roles of the contemporary and past forests by demonstrating that all four diversity metrics decline with increasing distance away from our approximations of forest refugia. This finding highlights the importance of considering deep‐time processes in modern conservation strategies.

### 4.1 Contemporary Climate and Forest

Most studies investigating the effects of contemporary and past climate on species diversity are biased toward favoring contemporary variables because of the high resolution of current climate data and diversity metrics in contrast to coarse past data (Araújo et al. 2008). As more studies evaluate equivalently scaled datasets, the influence of contemporary climate is often equally good or inferior to climate stability for predicting diversity (Araújo et al. 2008; Colville et al. 2020; Pinkert et al. 2020). In our study, contemporary mean annual temperature explained little of the variation and was negatively correlated with all four diversity metrics (Tables 1 and 2). Previous studies also found temperature to be negatively correlated with PE in frogs at the regional scale in East Africa (Barratt et al. 2017) and continental scale across Africa (Pinkert et al. 2020). In the LGF, temperature is relatively uniform across the landscape, which could explain its weak predictive power. The lower temperatures at high elevations in the Cameroon Volcanic Line, which hosts high species diversity, likely explain the negative correlation of overall diversity with temperature. We found that mean annual precipitation was a strong, positive predictor of diversity, second only to forest stability (Table 1). Pinkert et al. (2020) found a similar relationship between precipitation and species richness of frogs and, to a lesser extent, birds and mammals. This is a predictable pattern for frogs given their physiology and life history requirements related to water (Wells 2010).

### 4.2 Climate Stability

Comparative studies have increasingly used paleoclimatic data to generate quantitative estimates of past climate stability. Most studies quantify climate stability as the anomaly between contemporary climate and one or more past time periods (Jansson 2003; Colville et al. 2020). In our study, neither variable representing climate stability (temperature, precipitation) was effective at explaining contemporary afrobatrachian frog diversity metrics in the LGF. This is in line with results from East Africa, where both temperature and precipitation stability since the last interglacial (120 kyr) were negatively correlated with PE for other frog species (Barratt et al. 2017). This is likely because frogs have strict physiological requirements for which stability estimates do not account. For example, in this study, part of the Sudan Grassland region has been extremely stable for dry and hot conditions since the Pleistocene (Figure S3), but this stable climate is not conducive to supporting high species richness in amphibians. With stability analyses, two areas with opposite conditions can rank as equally stable, thus leading to poor predictive power overall of stability at a regional scale for taxa with known physiological constraints. For this reason, our approach of creating a summary of past environmental suitability based on ENMs of focal taxa provides a means to create quantitative estimates of the locations of refugia.

### 4.3 Forest Stability

For nearly a century, areas of forest stability (i.e., refugia) have been invoked to explain patterns of diversity in Africa (Lönnberg 1929; Chapin 1932). Although authors have utilized different approaches for delimiting stable refugia (palynology: Livingstone 1975; endemism: Prigogine 1988; paleoprecipitation: Anhuf et al. 2006), biogeographers have relied largely on binary (presence‐absence) delimitations to interpret patterns of diversification. Using ENMs for 10 forest‐obligate frog species and projecting their potential distributions across 257 time‐slices, we established the first quantitative estimate of forest stability since the Pleistocene for the LGF. Our stability map is largely congruent with previous delimitations of refugia based on fossil pollen (Maley 1996) and endemism (Carcasson 1964; Robbrecht 1996; Rietkerk et al. 1995) and is consistent with these other lines of evidence (endemism, palynology, paleoprecipitation) that the forest cover in the LGF has been more stable than the surrounding areas (Endler 1982; Maley 1996; Anhuf et al. 2006). Our surface stability maps provided no support for the Congo Fluvial Refugium proposed by Colyn (1991) (Figure 1B) which was based on the distribution of primate subspecies. However, this could be due to different physiological requirements for frogs in comparison to primates and, potentially, a different community of frog species in the central Congo Basin. This discrepancy highlights the value of extending ENM‐based stability analyses to other taxa with different physiological demands. To date, only a handful of studies have quantified habitat stability to test the contemporary and historical influences on diversity (Graham et al. 2006; Rosauer et al. 2015; Barratt et al. 2017). These studies reveal that habitat stability is equally or more important in explaining variation in species diversity. Barratt et al. (2017) similarly found that habitat stability was a strong predictor of frog endemism in the coastal forests of East Africa. Using recently developed paleoclimate models (Gamisch 2019), we probe deeper in time than previous studies. Our results add support to the importance of incorporating habitat stability—and not only climate stability—when comparing the roles of past and contemporary variables on diversity.

### 4.4 Distance From Refugia and Conservation

The ecological climate hypothesis accepts that past climates can leave signatures on contemporary patterns of species diversity, but proposes that species distributions and diversity are close to equilibrium with contemporary conditions (Hawkins et al. 2003). Under this scenario, we expect species diversity to be equally distributed across the current rainforest cover in the LGF. However, we found that SR, PD, and RPD decline with increasing distance from the nearest forest refugia, even across large regions of contemporary forest cover. This lends strong support for the idea that past stability is important to explaining patterns of diversity in the tropics. Diversity is, on average, higher in protected areas within stably forested regions (37–44 species/cell) than in protected areas overall in the region (23 species/cell). In the context of global efforts to expand protected area coverage, including the 30 × 30 target (Convention on Biological Diversity, 2022), these results highlight opportunities to prioritize future conservation actions toward regions that combine long‐term forest stability with high species richness and phylogenetic diversity. Expanding existing protected areas (e.g., Ebo Wildlife Reserve and Mount Manengouba National Park in Cameroon; Birougou National Park in Gabon) or creating new protected areas to maximize the amount of historically stable areas under protection can help to increase the number of frog species conserved. Central Cameroon has one of the largest continuous swaths of stable forest habitat with some of the highest species richness in the region; however, it currently has no legal protection (Figure S6) making it an important candidate for conservation efforts. Prioritizing historically stable areas could be crucial for conserving species in the face of future climate change (Michalak et al. 2018).

## 5 Conclusion

Our study supports that past forest stability best explains contemporary patterns of frog diversity in the Lower Guinean Forests, a biodiversity hotspot. Because fossil pollen records across this region lack the spatial and temporal resolution necessary for testing processes before the Holocene (Livingstone 2001), we relied on ENMs and paleoclimate models to produce a quantitative habitat stability map for forests in the region. The resulting map is a powerful tool for statistically testing the role of historical processes, namely fluctuating forest cover, on contemporary patterns of species diversity. Such surface stability maps can be extended to different taxa with divergent physiological requirements and shed light on the influence of stability in different habitats across taxonomic groups. Quantitative habitat stability maps may also elucidate evolutionary processes that generate genomic and functional diversity.

## Author Contributions

**Gregory F. M. Jongsma:** conceptualization (lead), data curation (lead), formal analysis (lead), funding acquisition (supporting), investigation (lead), methodology (equal), writing – original draft (lead). **Narayani Barve:** methodology (equal), resources (equal), software (equal), validation (equal), writing – review and editing (equal). **Julie M. Allen:** conceptualization (supporting), methodology (equal), resources (equal), software (equal), writing – review and editing (equal). **Hannah L. Owens:** methodology (equal), validation (equal), writing – review and editing (equal). **David C. Blackburn:** conceptualization (supporting), funding acquisition (lead), resources (equal), supervision (lead), writing – review and editing (equal).

## Conflicts of Interest

The authors declare no conflicts of interest.

## Supporting information


**Data S1:** ece373207‐sup‐0001‐Supinfo.docx.

## Data Availability

Data supporting the results of this study and stability map layers are archived in the Open Science Framework repository (https://doi.org/10.17605/OSF.IO/84B9P). All occurrence records used in this study are publicly available through the Global Biodiversity Information Facility (GBIF) as an occurrence download (https://doi.org/10.15468/dl.9fbrbr).
