# Supplementary material for: Pleistocene Forest Stability Predicts Patterns of Frog Diversity in Central Africa
Source: Ecol Evol. 2026 Mar 11;16(3):e73207. doi: 10.1002/ece3.73207 (PMC13093375; doi:10.1002/ece3.73207)
Supplement: Supplementary file 1 — Data S1: ece373207‐sup‐0001‐Supinfo.docx. [file ECE3-16-e73207-s001.docx]

SUPPORTING MATERIAL:

Pleistocene Forest Stability Predicts Patterns of Frog Diversity in Central Africa

***Phylogenetic Methods***

*16S Sequence*

Genomic DNA was extracted following standard procedures for Qiagen DNeasy kits. Sequence data were obtained from the mitochondrial marker *16S* ribosomal RNA using primers 16SA-l and 16SB-h (Palumbi et al. 1991). We performed polymerase chain reactions (PCRs) using a 20ul volume consisting of 2ul DNA and AmpliTaq Gold master mix. The amplification process was as follows: denaturation at 95°C for 10 minutes, then 35 cycles of 96°C for three seconds, 54°C for three seconds, 68°C for 15 seconds, and a final extension at 72°C for 10 seconds. We sent the resulting PCR product to Genewiz (Pennsylvania). Specimens and associated tissues (liver; Table S1) were collected and stored in RNAlater with permission from the Republic of the Congo under the permit N°134 /MRSIT/IRSEN/DG/DS and exported under N°135 /MRSIT/IRSEN/DG/DS.

*Phylogenetic Tree*

To explore patterns of phylogenetic diversity of Afrobatrachian frogs, we modified a published dataset (Portik et al. 2019). We trimmed any tips not represented in our study region (upper left: 12° N, 7° E; lower right: 10° S, 20° E). We added some taxa not included in the original phylogeny (Table S1). Three taxa, which do not have any associated DNA sequence (*Leptodactylodon blanci*, *L. stevarti*, *Hyperolius bopeleti*) were added to the phylogeny using the R package *add.tip* function in the ‘phylotools’ R package. The position in the tree was based on their hypothesized relationships (*L. blanci*: Ohler, 1999; *L. stevarti*: Rödel & Pauwels, 2003; *H. bopeleti*: Amiet, 2012). Portik et al. (2019) inferred a species tree based on 1047 exons (561,180 base pairs). To capture a maximum number of species, we created a tree based on *16S* ribosomal DNA. To preserve the strongly supported relationships inferred in Portik et al.’s (2019) larger dataset, we used this phylogeny as a constraint tree (-g function) for our maximum likelihood analysis in RaxML v.8 (GTR+I+G; 10,000 ultrafast bootstraps). We converted the ML tree to a chronogram using the *chronopl* function in the R package, ‘ape’, with 65 time calibrations based on the Portik et al. (2019) tree. A list of species from the Lower Guinean Forest that were added to the Portik et al. (2019) phylogeny can be found in Table S1.

***Ecological Niche Models***

*Occurrence Data*

Frogs are excellent taxa for tracking historical shifts in vegetation in response to climate change because they are poor dispersers and have strict habitat requirements (Zeisset & Beebee, 2008). To quantify historical forest stability (refugia), we modeled 10 co-distributed, forest-obligate frog species across western equatorial Africa during the Pleistocene (2.58 myr – 20 kyr). For each species, we compiled records from GBIF and iDigBio databases using the R package, ‘spocc’ (Chamberlain et al. 2016), and from our fieldwork. We filtered occurrence data in a stepwise fashion to improve quality. First, using the R package, ‘scrubr’ (Chamberlain et al. 2016) records with absent or spurious GPS coordinates were removed. Next, we removed all duplicate coordinates. We visually inspected the records by projecting them on a map of Central Africa and their IUCN distribution. Records commonly fall outside of the IUCN distributions, however, records beyond 100 km from its IUCN delimits were only accepted if their identification was confirmed by an expert, photos, or molecular barcodes (all data is available on Open Science Foundation; ​​DOI 10.17605/OSF.IO/84B9P). Occurrence records used in this study are publicly available through the Global Biodiversity Information Facility (GBIF) as an occurrence download (<https://doi.org/10.15468/dl.9fbrbr>).

*Environmental Data: past and present*

We used the bioclimatic variables from the WorldClim data repository (v1.2; [www.worldclim.org](http://www.worldclim.org)), soil moisture-retention (v%; data.isric.org), and slope. We cropped all the world raster files to Lower Guinean Forest and surrounding area (upper left: 12° N, 7° E; lower right: 10° S, 20° E) and removed variables that had a correlation coefficient of >90%, using the *pairs* function in R v3.5.3. This resulted in nine independent variables (Bioclim 1, 4, 6, 7, 12, 16, 17, soil moisture, and slope) to predict species distributions. To predict areas of forest stability in the past we used the same bioclimatic variables from the Oscillayers data repository (<https://doi.org/10.5061/dryad.27f8s90>) for 257 timeslices (roughly 9,375 year-intervals) going back 2.58 million years into the past. Slope and soil moisture-retention layers were constant across time slices.

*Ecological Niche Modeling*

Contemporary Ecological Niche Models (ENMs) were estimated for 80 species of Afrobatrachian frogs and for 10 non-Afrobatrachian frog species (Table S1). We used the R package, ‘kuenm’ (Cobos et al. 2019) to thin our records by 10 km distance to minimize record clustering, which can lead to biased predictions (Boria et al. 2014). The datasets used to develop each species’ model are available on Open Science Foundation (​​DOI 10.17605/OSF.IO/84B9P). To delineate a realistic calibration region (M region; Barve et al. 2011) where a species is likely to be found within, we used an IUCN range map buffered by 100 km (<https://www.iucnredlist.org/>). If vetted records were found outside of the IUCN range maps, we modified the polygons using QGIS v3.6.

To estimate environmental stability during the Pleistocene (2.58 myr ‐ 20 kyr), we used the R package ‘kuenm’ (Cobos et al. 2019) to calibrate, project, and evaluate each model. Models were calibrated using the maximum entropy machine learning algorithm Maxent ( v.3.4; Phillips et al. 2006). For each species, we generated a suite of candidate models with unique regularization multiplier (0.1–1.0 at intervals of 0.1, 2–6 at intervals of 1, and 8 and 10) and combinations of three feature class parameters (linear = l, quadratic = q, product = p; Cobos et al. 2019). The best models were selected based on significant partial ROC and AIC scores (Peterson et al. 2008; 10 bootstrap replicates, E = 5%). To limit overprediction, we did not permit extrapolation in the model projections onto past time slices. To convert raw Maxent outputs into presence/absence suitability maps for each species, a 5% minimum occurrence point threshold value was selected based on the 5th percentile suitability score as species’ occurrences used in the model calibration

***Tables***

Table S1: List of species from the Lower Guinean Forest added to Portik et al. (2019) phylogeny. UF specimens are from the Florida Museum of Natural History’s herpetology collection.

| Species | GenBank/Museum # |
| --- | --- |
| *Arthroleptis carquejai* | UF-180463 |
| *Arthroleptis xenochirus* | MK464474 |
| *Hemisus guineensis* | MK036501 |
| *Hemisus perreti* | UF-180509 |
| *Cardioglossa congolia* | KR349248 |
| *Cardioglossa gratiosa* | EF641001 |
| *Astylosternus fallax* | MK318856 |
| *Astylosternus laurenti* | MK318856 |
| *Astylosternus montanus* | MK318863 |
| *Astylosternus perreti* | MK318870 |
| *Astylosternus ranoides* | MK318874 |
| *Astylosternus rheophilus* | MK318894 |
| *Hyperolius schoutedeni* | MK544960 |
| *Alexteroon obstetricans* | DQ283171 |

Table S2: Species used for creating habitat stability maps.

| **Species** | Habitat | Microhabitat | Breeding habitat | References |
| --- | --- | --- | --- | --- |
| *Amnirana albolabris* | Forest | Arboreal | Lentic/Lotic | Channing & Rödel, 2019 |
| *Amnirana amnicola* | Forest | Arboreal | Lentic | Channing & Rödel, 2019 |
| *Amnirana lepus* | Forest | Arboreal | Lotic | Channing & Rödel, 2019 |
| *Chiromantis rufescens* | Forest | Arboreal | Lentic | Channing & Rödel, 2019 |
| *Conraua crassipes* | Forest | Aquatic | Lotic | Channing & Rödel, 2019 |
| *Phrynobatrachus africanus* | Forest | Terrestrial | Lentic | Channing & Rödel, 2019 |
| *Phrynobatrachus auritus* | Forest | Terrestrial | Lentic/Lotic | Channing & Rödel, 2019 |
| *Ptychadena aequiplicata* | Forest | Terrestrial | Terrestrial/Lentic | Channing & Rödel, 2019 |
| *Sclerophrys camerunensis* | Forest | Terrestrial | Lentic/Lotic | Channing & Rödel, 2019 |
| *Sclerophrys gracilipes* | Forest | Terrestrial | Lentic | Channing & Rödel, 2019 |

***Figures***


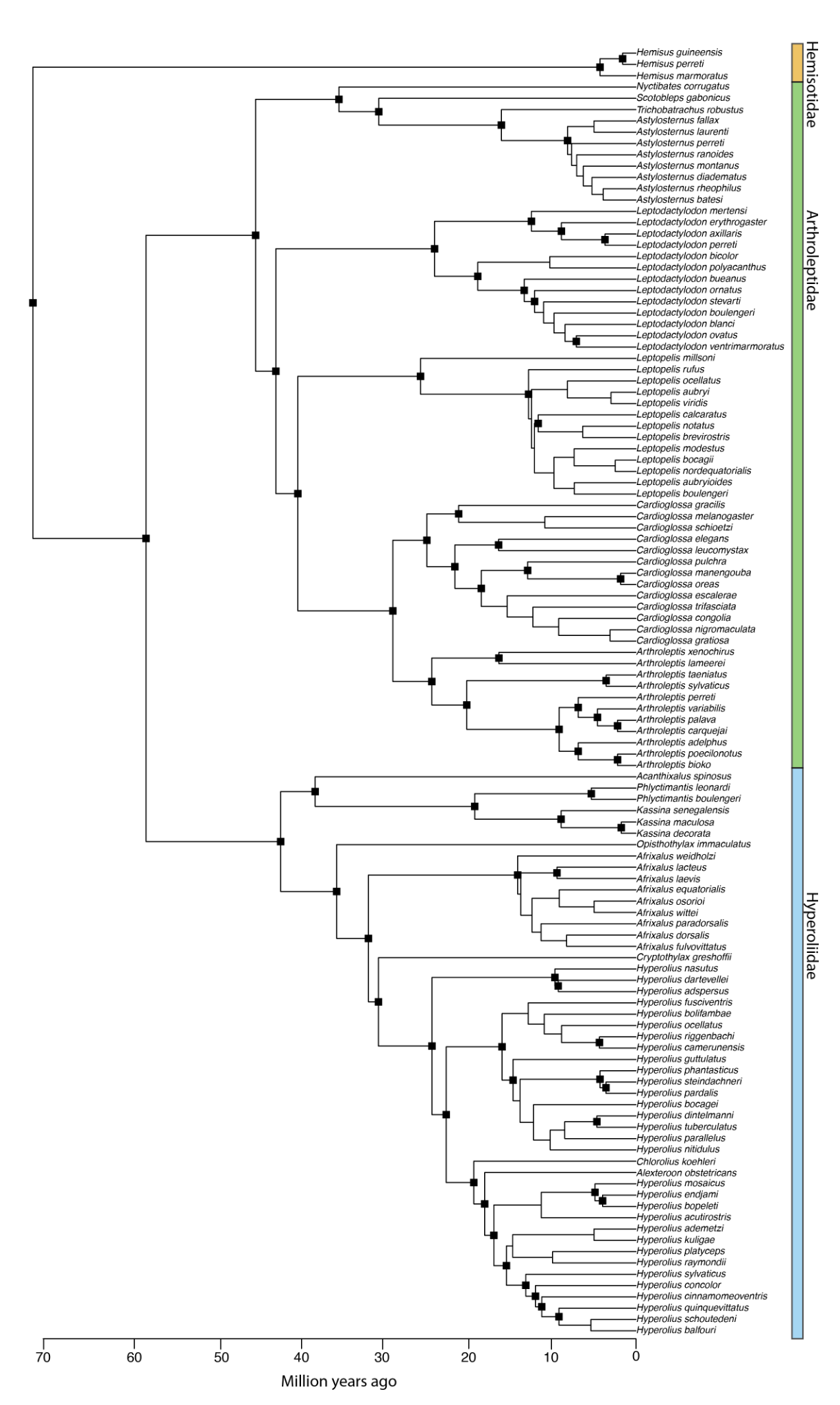


Fig. S1: Chronogram of Afrobatrachia found in the LGF. Black squares represent calibration points based on mean ages from Portik et al. (2019). Nexus tree available on OSF (DOI 10.17605/OSF.IO/84B9P).

***
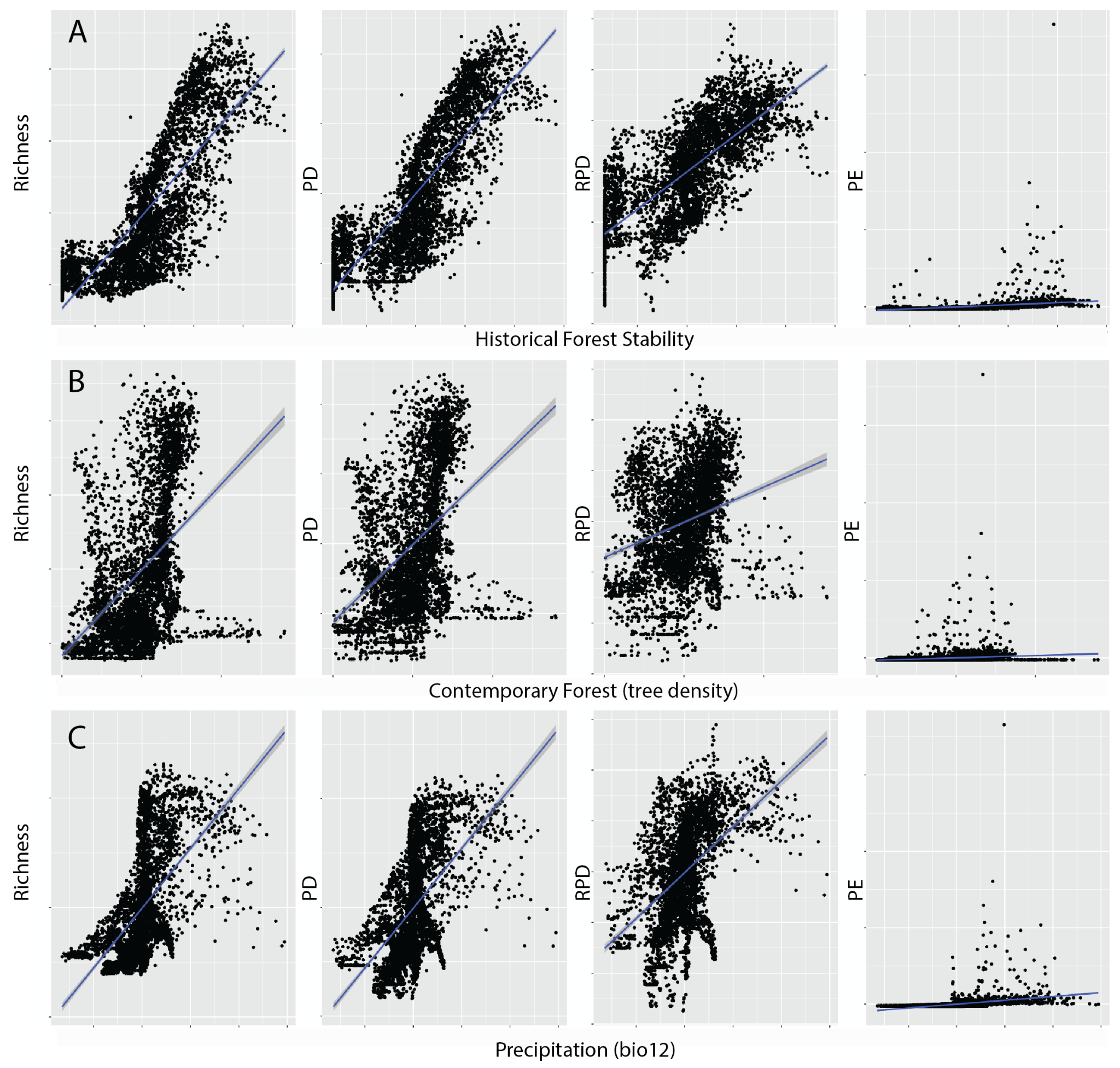
***

Fig. S2: Univariate linear models of richness, PD, RPD, and PE for forest stability (A), contemporary forest (B) and precipitation (C).

***Predictor Variables***


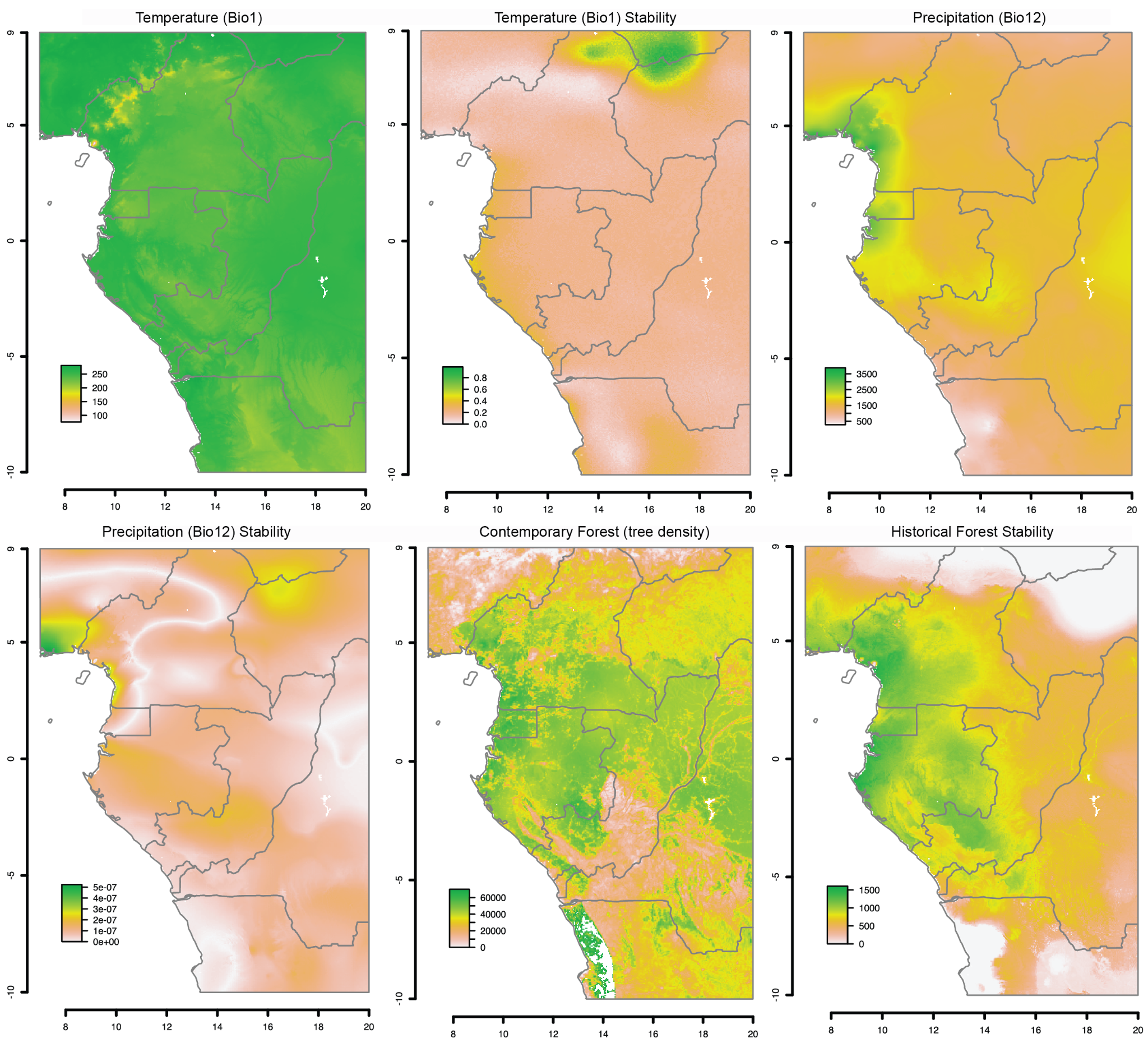


Fig. S3: Raw predictor values used to test patterns of diversity metrics (Richness, PD, RPD, PE). Rasters are available on OSF (DOI 10.17605/OSF.IO/84B9P)

***Thresheld Refugia***


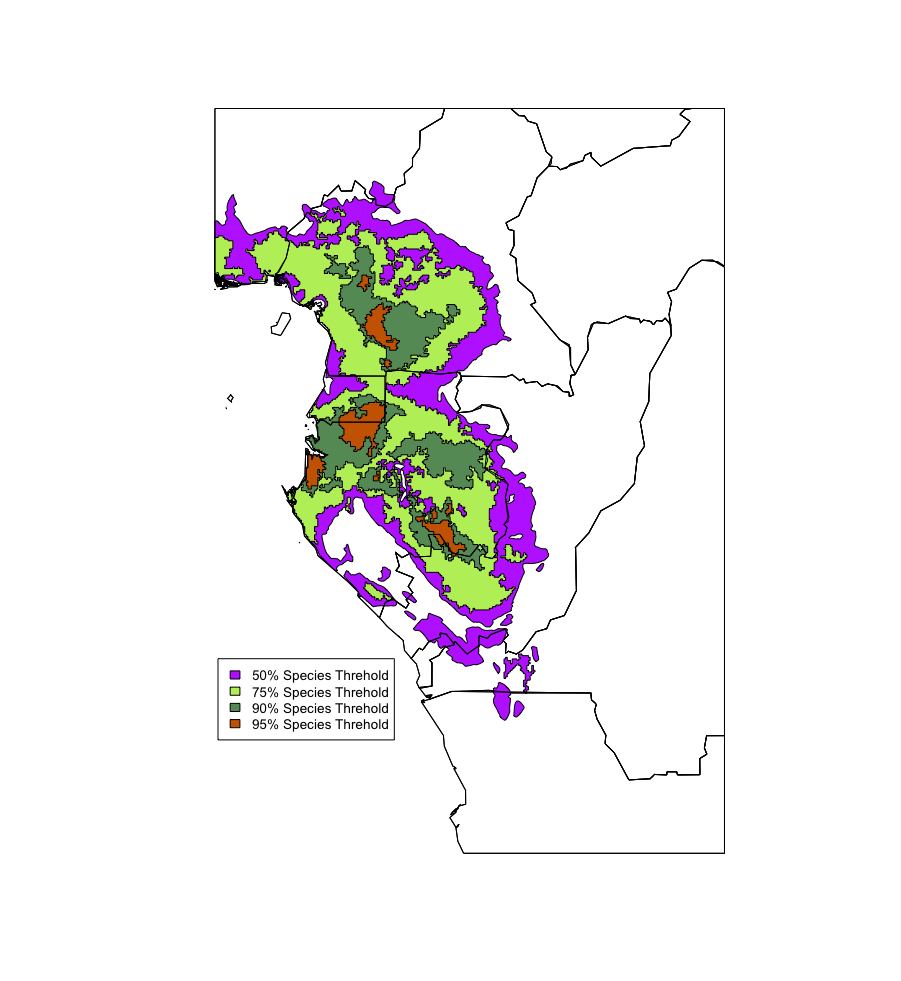


Fig. S4: We thresholded our stability map to create qualitative delimitations of stability, which we consider “refugia”. The strictest refugia were defined as having 95% of species present across 230 or more time-slices, and again for 90%, 75%, and 50% of species present. Each cell that met a specific threshold was converted to ‘one’ and cells that did not were converted to ‘zero’. Shapefiles are available on OSF (DOI 10.17605/OSF.IO/84B9P).

***Protected Areas in Stable Regions***


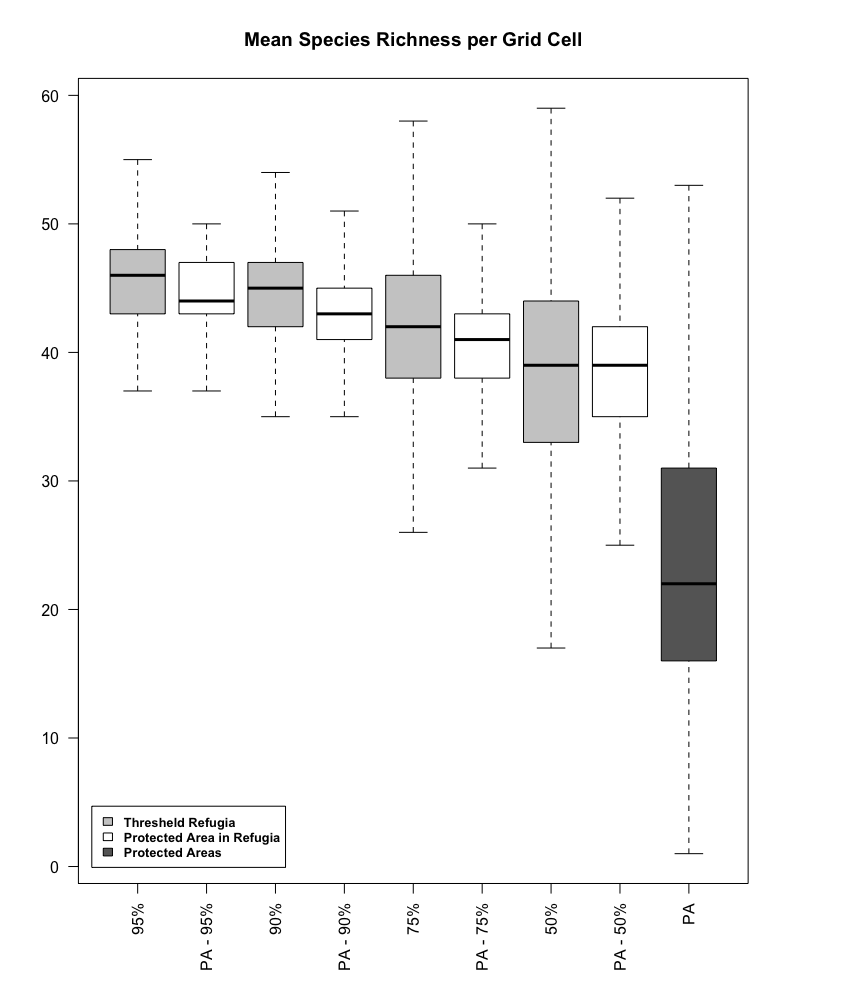


Fig. S5: Average species richness per cell in thesholded refugia (grey), protected areas within these refugia (white), and in all protected areas in the region (dark grey). Average species richness is higher in historically stable forest refugia than protected areas.

***Protected Areas and Stability***


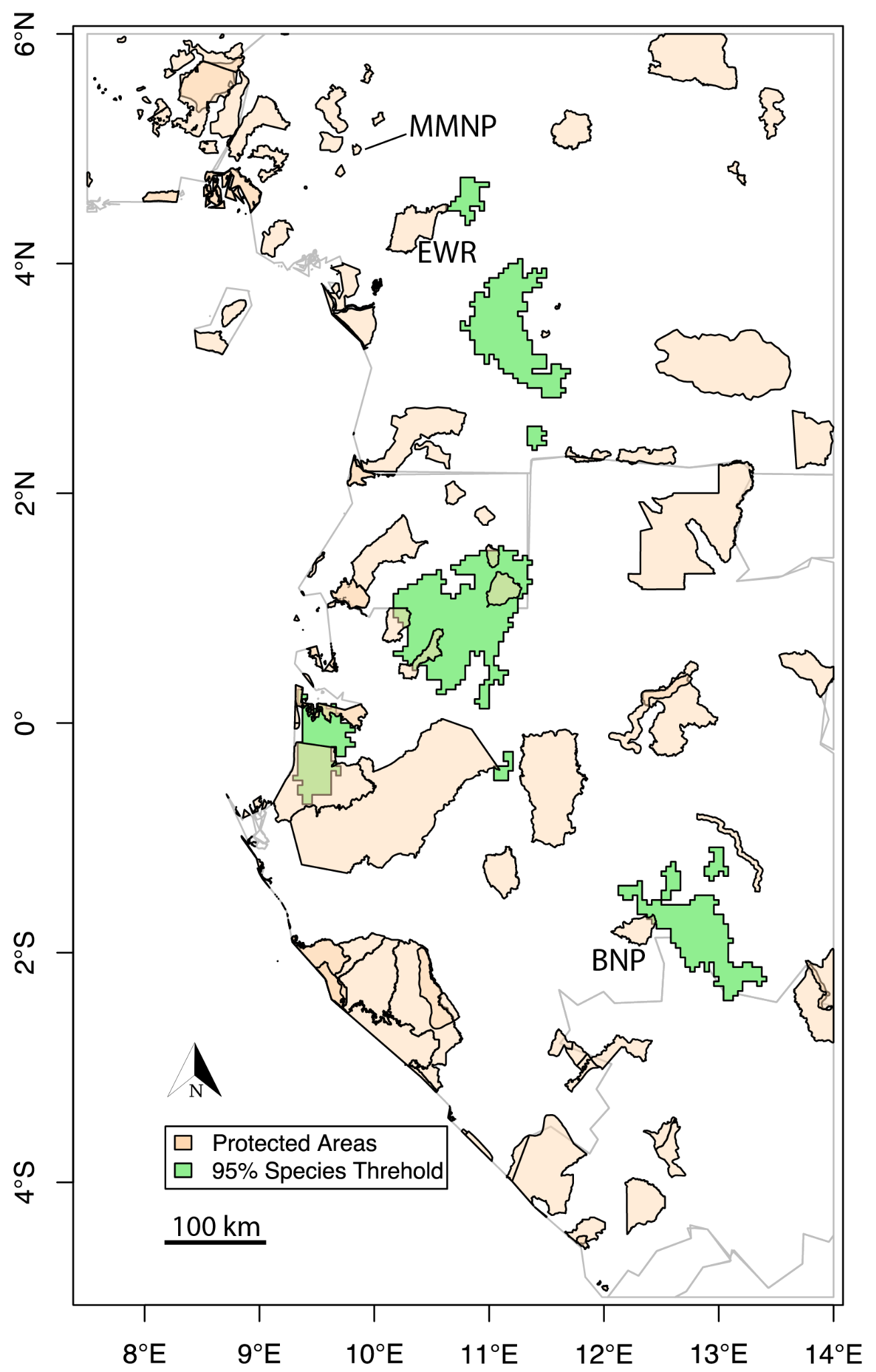


Fig. S6: Map of 95% thresholded stable refugia (green) and protected areas (beige). Mont Manengouba National Park (MMNP) is located in a center of endemism. Ebo Wildlife Reserve (EWR) overlaps slightly at its most easterly point with a refugium and would benefit from a northeastern expansion. Birougou National Park (BNP) overlaps slightly with a large refugium in the Chaillu Massif and would benefit from an eastern expansion. Lastly, there is a large refugium in south-central Cameroon that is entirely unprotected and should be made a priority for conservation efforts in the region.
